# Gene Expression in Aphelid Zoospores Reveals Their Transcriptional and Translational Activity and Alacrity for Invasion

**DOI:** 10.3390/jof11010068

**Published:** 2025-01-16

**Authors:** Igor R. Pozdnyakov, Alexei O. Seliuk, Kristina O. Barzasekova, Sergey A. Karpov

**Affiliations:** 1 Zoological Institute, Russian Academy of Sciences, St. Petersburg 199034, Russia; sergei.karpov@zin.ru; 2 Department of Invertebrate Zoology, Faculty of Biology, St. Petersburg University, St. Petersburg 199034, Russia; aleksey1seluk@gmail.com; 3 Bioengineering, Center for Chemical Engineering, ITMO University, St. Petersburg 197101, Russia

**Keywords:** aphelida, zoospores, gene expression, dispersal stage, opisthokonta

## Abstract

In *Aphelidium insulamus* (Opisthokonta, Aphelida) zoospores, the expression of 7708 genes out of 7802 described genes was detected. For 589 of them, expression levels were shown to be more than 10 times higher than the median level. Among the highly expressed genes with known functions, the largest functional categories were “Cellular Metabolism”, “Protein Synthesis”, “Cell State Control”, and “Nucleic Acid Processing”. Unlike fungal zoospores, translational and transcriptional activity was demonstrated for *A. insulamus* zoospores. With increasing temperature, the expression of many zoospore genes changed dramatically; the expression of heat shock and chaperone protein genes multiplied more than 30 times, indicating the high sensitivity of aphelid zoospores and their response to environmental changes.

## 1. Introduction

Aphelids are intracellular parasites of algae that belong to the lineage Holomycota in the super-group Opisthokonta [1]. They represent sister lineage to all «true» (osmotrophic) fungi [2]. According to modern taxonomy, aphelids, despite the fact of their type of feeding (phagotrophic, instead of osmotrophic), belong to Early Divergent Fungi (EDF) as a subkingdom Aphelidiomycete containing a phylum Aphelidiomycota [3,4,5]. Contrariwise, the authors consider aphelids separate from fungi as the phylum Aphelida [5,6].

Indeed, the aphelid life cycle superficially resembles the life cycle of many zoosporic fungi (e.g., Chytridiomycota, Monoblepharomycota, Blastocladiomycota, Sanchytriomycota) [5]. Zoospore production at the dispersal stage is a common trait in these groups, but aphelids have a phagotrophic intracellular stage (i.e., penetrating an algal cell and consuming its content by phagocytosis), compared to the sporangia of zoosporic fungi [2,6,7,8,9].

Aphelida is a poorly studied group. It consists of approximately 20 freshwater and two marine described species [10,11,12]. Several genomic and transcriptomic studies [2,8,9] are aimed mostly on the resolution of the aphelid phylogenetic position, which was finally resolved by [2]. However, we still know little about the biochemistry, physiology, and general metabolism of this group, which is crucial for understanding the origin and evolution of fungi. Just a few studies are dedicated to the comparison of some essential signaling proteins in aphelids and other opisthokonts [8,13], and gene expression profiles during zoosporogenesis in Aphelida and Blastocladiomycota [14].

Zoosporic fungi of the phyla Chytridiomycota and Blastocladiomycota are better studied in these terms. Besides common genome and transcriptome assemblages, several attempts have been made to analyze differential expression throughout the life cycle of some species of Chytridiomycota, such as *Batrachochytrium dendrobatidis* and *Rhizoclosmatium globosum* [15,16], and Blastocladiomycota, such as *Coelomomyces lativitattus* [17]. These analyses reveal numerous differentially expressed transcripts, but the majority do not have corresponding functional annotation. Thereby, these studies only superficially show the main characteristics of metabolism during life cycle stages, such as the upregulation of amino acid biosynthesis, carbon metabolism, and the tricarboxylic acid cycle during germling formation from settled zoospores. Transcriptional and translational activity is lacking in the zoospores of *Rhizophlyctis rozea* [18], *R. globosum* [14], *Blastocladiella emersonii* [19,20], and *C. lativitattus* [17] even though they use lipids and carbohydrates gained from their sporangial stage [21]. The ribosomes in zoospores of all these species, excluding *R. rozea*, aggregate into clusters, historically called “nuclear cap” or ribosomal core, containing maternal mRNA blocked at the elongation stage. Ribosomes of *R. rozea* zoospores lie freely in the cytoplasm [22]; nonetheless, protein synthesis is also blocked [18]. Therefore, whether fungal zoospores have aggregated ribosomes or not, they produce no mRNA or proteins, which only starts when zoospores settle and begin to encyst on substrate or host surfaces.

All aphelid zoospores studied so far lack ribosomal aggregations [23,24,25,26,27,28,29,30,31]. Here, we perform a transcriptomic analysis of the zoospore stage of *Aphelidium insulamus* (Opisthokonta, Aphelida) [26] to reveal the metabolic characters of aphelid zoospores. We demonstrate their distinctive transcriptional and translational activity compared to that of fungal zoospores.

## 2. Materials and Methods

The culture of *Aphelidium insulamus* is maintained in the culture collection of parasitic protists (CCPP) of the Zoological Institute Russian Academy of Sciences (ZIN RAS) [32]. For the experiment, the culture was maintained at room temperature. Four samples of zoospores were obtained by filtering the culture fluid during the mass release of zoospores. Filtration was performed through a 4-fold folded filter with a mesh size of 25 μm; the sediment was then washed from the filter. Microscopic inspection showed minimal presence of algae threads. Then, the filtered liquid was centrifuged at 10× *g*, the liquid was removed, and the sediment was frozen at −80 °C. The design of the experiment is based on the possibility of the bioinformatic separation of aphelid transcripts from the host ones. The cells of other aphelid stages, if accidentally collected together with algae, should be very rare and their transcripts will not have a significant effect on the number of transcripts of genes with high expression levels in zoospores. In addition, three zoospore samples were collected with a micromanipulator under a microscope and frozen at −80 °C in a 5 µL drop.

c-DNA was prepared, amplified, and fragmented using the NEBNext^®^ Single Cell/Low Input RNA Library Prep Kit for Illumina, NEB #E6420, via the Low Input protocol. The final preparation of sequencing libraries was performed using the NEBNext^®^ Multiplex Oligos for Illumina^®^, NEB #E7416, New England Biolabs, Ipswich, MA, USA. Libraries were sequenced on the Illumina platform to obtain 2 × 100 paired-end reads. The resulting raw read libraries were processed in a Trimmomacic 0.40 [33] to remove low-quality initial and end regions and then aligned to the predicted transcriptome of *A. insulamus* [8] in Bowtie2 2.5.4 [34].

Raw counts of aligned transcripts for each sample were made in eXpress 1.5.1 [35]. The batch effect correction was performed separately for the filtered zoospore samples and the zoospore samples collected with a micromanipulator in the python version of ComBat-seq included in the InMoose 0.7.3 package [36]. After correction, the RPM (reads per million) indicator was calculated for all genes in each sample. For each of the two groups of zoospores, the average RPMs were calculated for each gene, the median value of the average RPM was determined, and then the average RPM values were divided by the median value.

The gene expression level was assessed relative to the median value. For functional analysis, genes with expression levels more than 10 times higher than the median were selected. It can be expected that their expression values reflect the performance of the most vital functions of the zoospore and are not significantly affected by transcripts of parasitic stages accidentally acquired with algal cells. To assess differential gene expression between zoospores collected by two different methods, the mean value of raw counts for each gene in each of the two groups of zoospores was calculated. The analysis of the average values of two sample groups was performed using the python version of DESeq2 of the InMoose 0.7.3 package. The difference in expression level was considered significant if the *p* value was below 0.05. Genes with a 30-fold or greater increase in expression level were considered for functional analysis as controlling the most significant cellular responses.

Functional analysis of genes was performed based on gene annotation in Pfam, InterPro, EggNog, COG, and GO databases. For comparison, all selected genes were assigned a functional category in the COG terminology. As a result, the following categories (minor categories) were identified: “Amino acid transport and metabolism”; “Carbohydrate transport and metabolism”; “Cell cycle control, cell division, chromosome partitioning”; “Cell wall/membrane/envelope biogenesis”; “Chitin synthesis”; “Chromatin structure and dynamics”; “Coenzyme transport and metabolism”; “Cytoskeleton”; “Defense mechanisms”; “Energy production and conversion”; “Function unknown”; “Inorganic ion transport and metabolism”; “Intracellular trafficking, secretion, and vesicular transport”; “Lipid transport and metabolism”; “Nucleotide transport and metabolism”; “Posttranslational modification, protein turnover, chaperones”; “Replication, combination and repair”; “RNA processing and modification”; “Secondary metabolites biosynthesis, transport and catabolism”; “Signal transduction mechanisms”; “Transcription”; “Translation, ribosomal structure and biogenesis”. The category “Chitin Synthesis” was identified separately because of the special function of this process (see Section 4). Allocation was made based on full annotation. For proteins with no information about their functions in any of the databases, a search for homologues was performed in the BLAST system using the blastp tool [37]. Proteins with unknown functions for which fungal homologues were found were marked as specifically fungal. Those for which only aphelid homologues were found were marked as specific aphelid. If there were no matches in BLAST, the protein was considered specific for *A. insulamus*.

In order to identify the main vital tasks performed by the cell, the COG categories were combined into 8 larger categories (large categories): “Protein Synthesis”, “Cellular Metabolism”, “Cell State Control”, “Nucleic Acid Processing”, “Cytoskeleton”, “Defense Mechanisms”, “Membrane Biogenesis”, and “Chitin Synthesis”.

## 3. Results

In zoospores of *Aphelidium insulamus*, the expression of 7708 genes out of 7802 previously described genes of this organism [8] was detected. A total of 7012 genes showed expression levels (in RPM) in the range of 0.1 to 9.9 from the median, and of these, 3825 genes had expression levels that deviated from the median by no more than two times. For 589 genes, expression levels were more than 10 times higher than the median. We focused on the analysis of this set of genes, believing that it ensures the implementation of the most vital functions of the zoospore cell. Among these 589 genes, 56 showed expression levels more than 100 times higher than the median.

**Figure 1:**
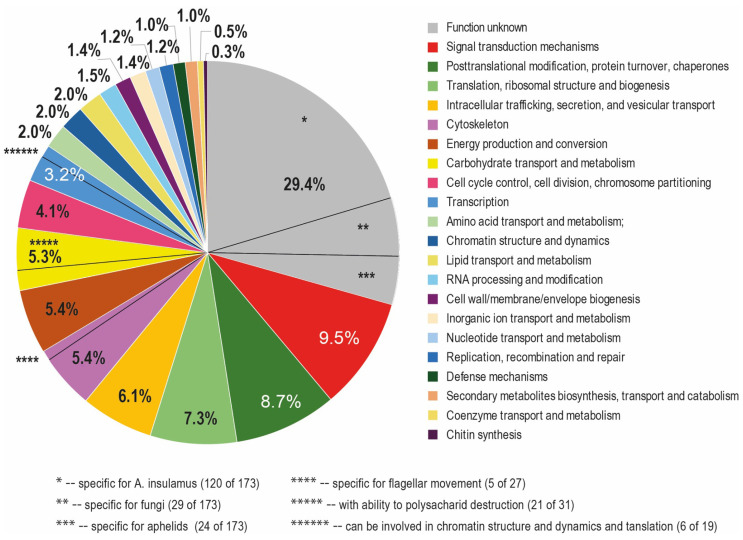
Percentages of genes of minor functional categories and subcategories among all genes with expression levels more than 10 times higher than the median.

The full list of genes with expression levels more than 10 times higher than the median, divided into 24 functional categories (minor categories), their annotations, and sources for annotations, are given in Supplementary Table S1. Table 1 shows the number of genes in each minor functional category and their percentages among all the genes analyzed. Figure 1 visualizes the percentages of genes.

According to the annotations, 29.4% (173) of genes with expression levels greater than 10 times the median are genes of unknown function. Of these, 20.4% (120) are specific to *A. insulamus*, 4.9% (29) are specific to fungi, and 4.1% (24) are specific to aphelids. The largest category of genes with known functions is the “Signal transduction” genes, accounting for 9.5%. It is followed by the “Post-translational modification, protein turnover, chaperones” category with 8.7%, and then the sizes of the categories decrease consistently. Among the genes in the “Carbohydrate transport and metabolism” category (5.3% of all), there is a large number (21 out of 31) of those whose products can be used for the degradation of polysaccharides. Of the 32 genes in the “Cytoskeleton” category (5.4% of all), 5 are directly involved in flagellar movement. In the group of 29 genes in the “Transcription” category, 6 genes have a broad spectrum of activity, including involvement in the regulation of chromatin structure and dynamics.

**Figure 2:**
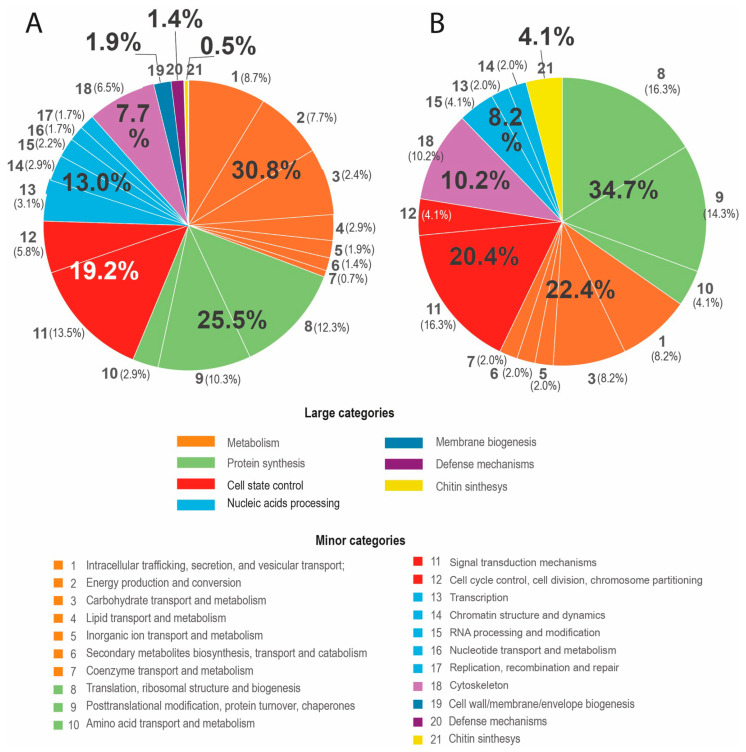
Percentage of genes in minor and major functional categories among genes with known functions, of those with expression levels greater than 10 times the median (**A**) and greater than 100 times the median (**B**). Large numbers with a percent sign are the share of a large functional category. Medium-sized numbers without a percent sign are the number of a small functional category. Small numbers with a percent sign in brackets are the share of a small functional category.

When selecting only the genes with known functions from all the genes expressed more than 10-fold and dividing selected genes into eight functional categories (large categories) (Figure 2A), it turns out that the majority of such genes (30.8%) belong to the “Cell metabolism” category. The next group (25.5%) consists of genes forming the “Protein synthesis” category. The third, fourth, and fifth categories are “Cell state control”, “Nucleic acid processing”, and “Cytoskeleton”, which account for 19.2%, 13.0%, and 7.7%, respectively. The remaining genes considered are classified as “Membrane biogenesis”, “Defense mechanisms”, and “Chitin synthesis” and have very small percentages, which are 1.9%, 1.4%, and 0.5%, respectively.

A selection of genes with expression more than 100 times than median shows that the same three functional categories dominate this narrow set, but their proportions are changed (Figure 2B). The share of “Protein synthesis” genes (34.7%) is the highest here. The second position is occupied by the genes of “Cellular metabolism” (27.4%), and the third position is still occupied by the category “Cell state control” (20.4%). The proportion of “Cytoskeleton” genes increased (fourth position, 10.2%), while the proportion of “Nucleic acid processing” genes became smaller (fifth position, 8.2%). The remaining 4.1% belonged to two “Chitin synthesis” genes.

In the micromanipulator-collected zoospores we found 3084 genes with altered expression levels compared to the filtered zoospores. Among them, 98 genes had an increase of the expression level that was greater than 30-fold. Supplementary Table S2 lists the 98 genes, divided into 24 categories according to COG, their annotations, and the sources for the annotations. Table 2 contains the number of genes in each minor functional category and their percentages among the given set of genes.

Among the reported genes with an expression greater than 30-fold, the proportion of genes with unknown expressions is again the highest (33.6%). They also include A. insulamus-specific genes, fungal-specific genes, and aphelid-specific genes. The next largest group of genes are the genes in the category “Post-translational modification, protein turnover, chaperones” (28.6%). All other categories include a much smaller number of genes, and their percentage within the considered group fluctuates between 5.1 and 1%.

**Figure 3:**
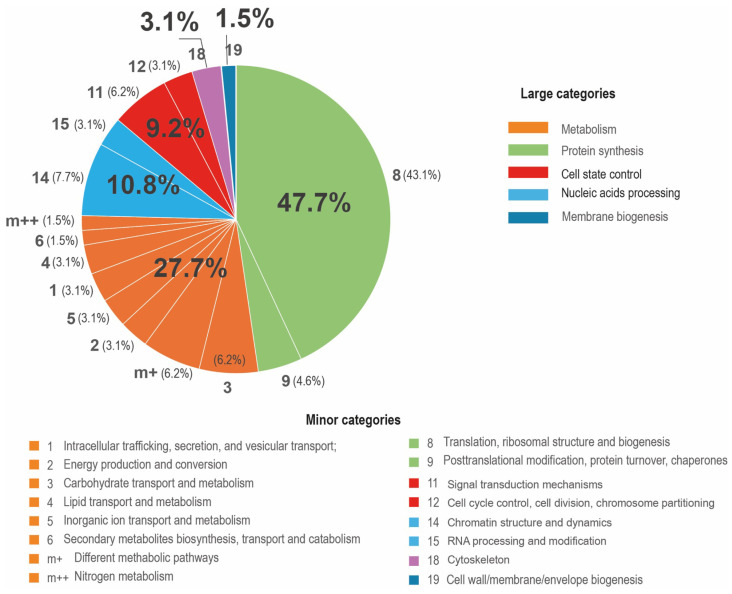
Percentage of genes in minor and major functional categories among genes with known functions, of those with expression levels magnified more than 30 times when collected with a micromanipulator. Large numbers with a percent sign are the share of a large functional category. Medium-sized numbers without a percent sign are the number of a small functional category. Small numbers with a percent sign in brackets are the share of a small functional category.

Figure 3 shows the percentage of genes in each large functional category among all genes with a more than 30-fold expression level. The genes controlling protein synthesis make up almost half of all genes (47.7%). The share of the next category, i.e., “Cellular Metabolism”, is almost two times smaller (27.7%). Next come “Nucleic acids processing” (10.8%) and “Cell state control” (9.2%). The smallest shares were found in the genes of the categories “Cytoskeleton” (3.1%) and “Membrane biogenesis” (1.5%).

According to the annotations, most genes associated with post-translation produce either proteins associated with heat shock proteins, heat shock proteins themselves, or other proteins with chaperone activity.

Seven genes have an extremely high expression level—more than 500 times. Their proteins are characterized by the following functions: one of them works in the proteasome, one participates in RNA processing, one is a heat shock protein, one is an activator of heat shock protein, one occurs in chromatin structuring, one exhibits chaperone activity, and the function of last one is unknown.

## 4. Discussion

Gene expression data clearly show that the zoospore of *A. insulamus* is a very metabolically active cell with extensive protein synthesis. In addition to protein synthesis genes, genes of cellular metabolism, including energy production, as well as genes controlling the cell state have significant shares among the highly expressed genes. Apparently, the main task of the aphelid zoospore is the synthesis of proteins, which serve to all other tasks, maintaining the active life of cells and quickly switching cell states in response to circumstances. This is entirely consistent with everything known at present about aphelid biology.

Active protein synthesis indicates that the proteins required for the zoospore are not retained from the previous life cycle stage (plasmodium) but are actively emerging during the life of the zoospore. These data do not coincide with the information on the translation absence in fungal zoospores [15,17,18,19,20], but they are in agreement with noted lack of ribosome aggregation in aphelid zoospores. It is noteworthy that among the genes with the highest expression levels, genes controlling protein synthesis occupy first place in quantity. This emphasizes their exceptional importance for the zoospore.

In *A. insulamus* zoospores, we found a pool of highly expressed genes with function related to nucleic acid processing including transcription. Genes involved in the regulation of chromatin structure may also be involved in transcription-related processes. Thus, it can be concluded that not only translation but also transcription occurs in *A. insulamus* zoospores. This also does not correspond to what is known for fungal zoospores. It is possible that the observed difference between aphelid and fungal zoospores reflects a general difference between these taxa. Perhaps, fungal zoospores are more specialized than aphelid ones, and aphelid zoospores retain more features of the ancestral stage, when the flagellated phase was free-living and feeding, rather than simply dispersing [9]. Alternatively, the features of the studied fungal and *A. insulamus* zoospores may not reflect the features of all members of the phyla but may be specific to the studied species. For example, it may define the lifespan of zoospores.

Among the actively expressed *A. insulamus* genes are two genes associated with chitin synthesis. Chitin synthesis does not occur at the zoospore stage, but is necessary for the next stage, formation of the cyst. Active expression of chitin synthesis genes shows that the zoospore is already prepared for a quick transition to the cyst stage. Notably, genes associated with chitin synthesis are among the genes with the highest (more than 100 times higher than the median) expression level. The proteins associated with polysaccharide degradation can also be used to destroy the host cell wall.

Gene expression differed drastically in zoospores collected with a micromanipulator. Activity increased for some genes and decreased for others. Heating during their isolation in a microvolume of water and their exposure to lamp light are the most likely causes of this change in expression. Apparently, the cell tries to protect itself by changing all areas of metabolic activity. Approximately 50% of the genes associated with protein synthesis, mainly the heat shock proteins, as well as the synthesis of other proteins with chaperone activity that provide correct protein folding, had higher expression levels. This reflects the critical task of the cell to maintain the working condition of the protein synthesis system. Second most important are the genes that affect metabolism, which is obviously changing during these conditions. The expression of some genes associated with nucleic acid processing also increases significantly. Among them are genes controlling the structure of chromatin predominate, which apparently reflects the need to protect chromatin stability under heat stress conditions.

The observed changes in cellular state indicate the high sensitivity of zoospores and their ability to quickly respond to environmental changes. It also further confirms the antiquity and versatility of the defense system based on heat-shock proteins.

## Figures and Tables

**Table 1:** The gene numbers in each minor functional category and subcategories of genes with expression levels more than 10 times higher than the median and their percentages among all genes analyzed.

Functional Category	Subcategory	Number	Percentage
Function unknown	Specific for *A. insulamus*	120	20.4%
	Specific for fungi	29	4.9%
	Specific for aphelids	24	4.1%
	Total	173	29.4%
Signal transduction mechanisms		56	9.5%
Posttranslational modification, protein turnover, chaperones		51	8.7%
Translation, ribosomal structure, and biogenesis		43	7.3%
Intracellular trafficking, secretion, and vesicular transport		36	6.1%
Cytoskeleton	Specific for flagellar movement	5	0.8%
	Other	27	4.6%
	Total	32	5.4%
Energy production and conversion		32	5.4%
Carbohydrate transport and metabolism	Ability to cause polysaccharide destruction	21	3.6%
	Not involved in polysaccharide destruction	10	1.7%
	Total	31	5.3%
Cell cycle control, cell division, chromosome partitioning, and cytoskeleton		24	4.1%
Transcription	Only	13	2.2%
	Additionally: chromatin structure and dynamics; translation, ribosomal structure, and biogenesis	6	1.0%
	Total	19	3.2%
Amino acid transport and metabolism;		12	2.0%
Chromatin structure and dynamics		12	2.0%
Lipid transport and metabolism		12	2.0%
RNA processing and modification		9	1.5%
Cell wall/membrane/envelope biogenesis		8	1.4%
Inorganic ion transport and metabolism		8	1.4%
Nucleotide transport and metabolism		7	1.2%
Replication, recombination and repair		7	1.2%
Defense mechanisms		6	1.0%
Secondary metabolites biosynthesis, transport, and catabolism		6	1.0%
Coenzyme transport and metabolism		3	0.5%
Chitin synthesis		2	0.3%
Total		589	100.0%

**Table 2:** The gene numbers in each minor functional category and subcategories of genes with the expression level magnification greater than 30-fold when micromanipulator collected and their percentages among all genes analyzed.

Functional Category	Subcategory	Number	Percentage
Function unknown	Specific for *A. insulamus*	21	21.4%
	Specific for fungi	9	9.2%
	Specific for aphelids	3	3.1%
	total	33	33.7%
Posttranslational modification, protein turnover, chaperones		28	28.6%
Chromatin structure and dynamics		5	5.1%
Carbohydrate transport and metabolism. Polysaccharide degradation ability		4	4.1%
Different metabolic pathways		4	4.1%
Signal transduction mechanisms		4	4.1%
Translation, ribosomal structure and biogenesis		3	3.1%
Cytoskeleton		2	2.0%
Cell cycle control, cell division, chromosome partitioning		2	2.0%
Energy production and conversion		2	2.0%
Inorganic ion transport and metabolism		2	2.0%
Intracellular trafficking, secretion, and vesicular transport		2	2.0%
Lipid transport and metabolism		2	2.0%
RNA processing and modification		2	2.0%
Cell wall/membrane/envelope biogenesis		1	1.0%
Nitrogen metabolism		1	1.0%
Secondary metabolites biosynthesis, transport and catabolism		1	1.0%
Total		98	100.00%

## Data Availability

The original contributions presented in this study are included in the article/Supplementary Materials. Further inquiries can be directed to the corresponding author.

## Supplementary Materials

The following supporting information can be downloaded at: https://www.mdpi.com/article/10.3390/jof11010068/s1, Table S1: 589 genes with expression levels more than 10 times higher than the median and their division into 24 functional categories according to COG terminology; Table S2: 98 genes increasing of the expression level greater than 30-fold and their division into 24 functional categories according to COG terminology.
